# NPM1 and IDH1/2 Mutations Show Limited Prognostic Impact in Relapsed/Refractory AML: Evidence From the AVALON Cohort

**DOI:** 10.1002/hon.70169

**Published:** 2026-01-13

**Authors:** Calogero Vetro, Irene Azzali, Elisabetta Petracci, Cristina Papayannidis, Eleonora Eleuteri, Fanny Erika Palumbo, Vincenzo Federico, Nicola Fracchiolla, Patrizia Zappasodi, Maria Paola Martelli, Maria Benedetta Giannini, Lorenzo Brunetti, Raffaele Palmieri, Jacopo Nanni, Giorgia Simonetti, Fabio Guolo, Paola Minetto, Luca Maurillo, Federica Gigli, Atto Billio, Elisabetta Todisco, Giovanni Martinelli, Giovanni Marconi

**Affiliations:** ^1^ Hematology and Bone Marrow Transplantation Unit Hospital of Bolzano (SABES‐Azienda Sanitaria dell’Alto Adige) Teaching Hospital of Paracelsus Medical University Bolzano Italy; ^2^ IRCCS Istituto Romagnolo per lo Studio dei Tumori (IRST) “Dino Amadori” Meldola Italy; ^3^ Department of Hematology and Sciences Oncology Institute of Haematology “L. and A. Seràgnoli” S. Orsola University Hospital in Bologna Bologna Italy; ^4^ Division of Hematology A.O.U. Policlinico “G.Rodolico—S. Marco” Catania Italy; ^5^ Unità di Ematologia e TCS Ospedale “Vito Fazzi” Lecce Italy; ^6^ UOC Oncoematologia Fondazione IRCCS “Ca'Granda” Ospedale Maggiore Policlinico Milano Italy; ^7^ Dipartimento di Oncoematologia Fondazione IRCCS Policlinico “San Matteo” Pavia Italy; ^8^ Dipartimento di Medicina e Chirurgia Università di Perugia Ospedale “Santa Maria della Misericordia” Perugia Italy; ^9^ Department of Clinical and Molecular Sciences Università Politecnica delle Marche Ancona Italy; ^10^ Department of Biomedicine and Prevention University of Rome Tor Vergata Rome Italy; ^11^ Department of Internal Medicine Clinic of Hematology University of Genova Genova Italy; ^12^ UOSD Mieloproliferative Policlinico Tor Vergata Rome Italy; ^13^ Divisione di Oncoematologia IRCCS Istituto Europeo di Oncologia Milano Italy; ^14^ SC Ematologia Ospedale “Busto Arsizio” ASST Valle Olona Varese Italy; ^15^ UO Ematologia Università di Bologna Ospedale S. Maria delle Croci Ravenna Italy

## Abstract

In the AVALON cohort of relapsed/refractory AML treated with venetoclax plus hypomethylating agents, *NPM1* and *IDH1/2* mutations showed no significant impact on response or survival. These findings indicate that prognostic models for relapsed AML should consider treatment context rather than baseline mutation status.

To the Editor,

Venetoclax (VEN) combined with hypomethylating agents (HMAs) has changed the standard of care for newly diagnosed (ND) acute myeloid leukemia (AML) patients unfit for intensive chemotherapy [1, 2, 3]. In this setting, new prognostic models such as the Mayo Clinic model [4], the 4‐gene prognostic risk score (mPRS) [5], and the 2024 ELN classification [6] have refined molecular risk assessment under less‐intensive regimens. The mPRS links *TP53* to high risk and *NRAS*, *KRAS*, or *FLT3*‐ITD to intermediate risk, while ELN 2024 and the Mayo Clinic model emphasize the favorable role of *NPM1*, *IDH1*/*2*, and *DDX41* mutations.

By contrast, prognostic models in the relapsed/refractory (R/R) AML setting remain limited, with ELN22 and mPRS showing weak discriminating capabilities [6]. The recently proposed VENetoclax Prognostic Risk Score (VEN‐PRS), which integrates clinical and molecular factors in R/R AML treated with VEN + HMA, found no prognostic effect of *NPM1* and *IDH1/2* mutations [7].

To further explore this issue, we analyzed 147 VEN‐HMA treated R/R AML patients in the AVALON study [1]. Of them, 114 (78%) underwent molecular profiling: *IDH1* was tested in 50, *IDH2* in 56, *NPM1* in 91, while 33 (22%) remained untested. Profiling was more frequent among younger and heavily pretreated patients (median age 62 vs. 68 years; prior intensive therapy 91% vs. 17%). Allogeneic hematopoietic stem cell transplantation (HSCT) rates were comparable between tested and untested cases (28% vs. 18%, *p* = 0.36; details in Supporting Information S1: Table S1). Among tested patients, *IDH1, IDH2, and NPM1* mutations were detected in 6%, 18%, and 13% of cases, respectively (Supporting Information S1: Table S2). No differences in response, survival, or HSCT rates were observed. In the subgroup of 55 patients with complete *IDH1*, *IDH2*, and *NPM1* profiling, outcomes were comparable between those harboring ≥ 1 mutation (*n* = 25) and triple‐wild‐type cases (*n* = 30): ORR 41% versus 54% (*p* = 0.31), mDOR 6.5 versus 6.5 months (*p* = 0.46), mEFS 8.0 versus 8.9 months (*p* = 0.84), mOS 9.7 versus 8.9 months (*p* = 0.99), and HSCT rate 24% versus 37% (*p* = 0.31) (Table 1, Figure 1A–B). Conversely, analysis of ND patients from the AVALON study confirmed the favorable prognostic role of *NPM1* and *IDH1*/*2* mutations (Supporting Information S1: Tables S3–S5 and Figure S1). As expected, overall survival and event‐free survival were longer in ND rather than R/R patients (median OS 25.6 vs. 8.9 months, *p* = 0.02; EFS 20.1 vs. 8.9 months, *p* = 0.06, Supporting Information S1: Figure S2), reflecting the different disease settings and overall treatment expectations.

**TABLE 1:** Clinical outcomes by *NPM1*/*IDH1*/*IDH2* mutational status in R/R AML: Response, duration of response, event‐free survival, and overall survival in mutated versus triple‐wild‐type patients.

	NPM1/IDH		
	Wild type	Mutated	*p*
*n*.	30	25	
Best response—*n* (%)			
CR/CRp/CRi	11 (41)	8 (36)	
PR	1 (4)	5 (23)	
SD	14 (52)	8 (36)	
ED	1 (4)	1 (5)	
ORR^a^—*n* (%)	12 (41)	13 (54)	0.31
HSCT after VEN + HMA—*n* (%)	11 (37)	6 (24)	0.47
mDOR (months) [95% CI]	6.5 [2.0–13.2]	6.5 [3.0‐NR]	0.46
mEFS [95% CI]	8 [4–10.9]	8.9 [2.4–12.2]	0.84
mOS [95% CI]	9.7 [4.7–13.4]	8.9 [3.2–13.2]	0.99

Abbreviations: CR, complete remission; CRi, complete remission with incomplete reconstitution; CRp, complete remission with partial reconstitution; ED, early death (< 3 months without disease re‐evaluation); mDOR, median duration of response; mEFS, median Event free survival; mOS, median overall survival; NR, not reached; PR, partial response; 95% CI, 95% confidence intervals.

^a^ CR + CRp + CRi + PR.

**FIGURE 1:**
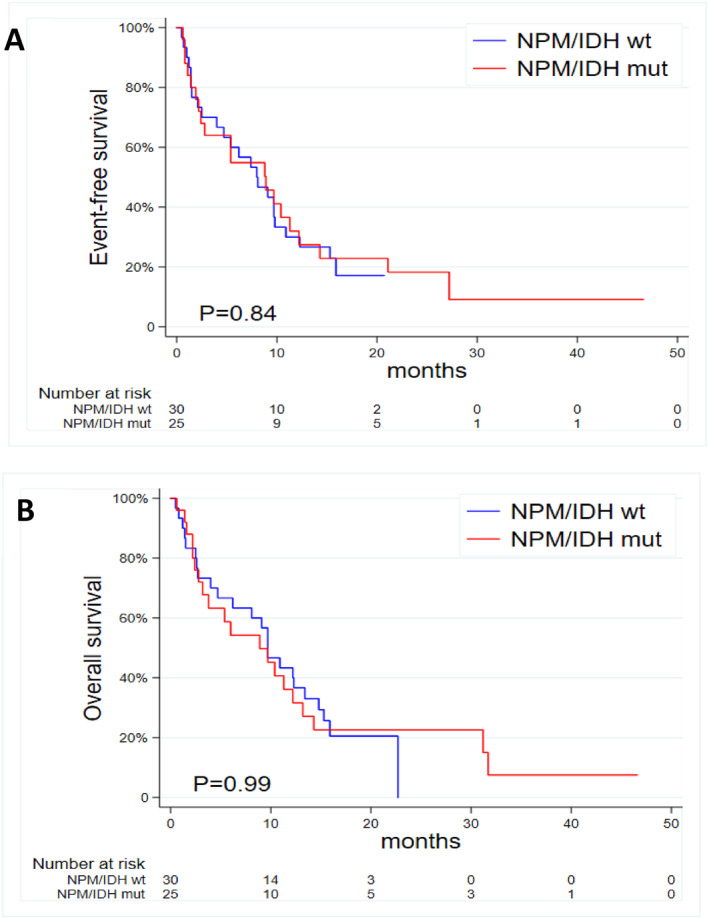
Event free survival (A) and overall survival (B) in R/R AML receiving VEN + HMAs comparing those with wild‐type *NPM1* and *IDH1/2* (triple‐wild‐type) versus those with at least one mutation in *NPM1*, *IDH1*, or *IDH2*.

The limited prognostic performance of molecular classifiers, such as mPRS, ELN22 in R/R setting highlights the need for dedicated risk models. Shahswar et al. analyzed 240 R/R AML patients treated with VEN‐based regimens, creating the first VEN + HMA‐specific prognostic model. Prior HMA exposure, extramedullary disease, and *TP53*, *FLT3*, *NF1*, or *PTPN11* mutations predicted poor outcomes, while *SF3B1* mutation correlated with better survival [7]. Patients were stratified into favorable (OS 21.4 months), intermediate (7.5 months), and adverse (4.6 months) groups. By outperforming ELN 2022 and Döhner's 4‐gene score, VEN‐PRS highlights limits of diagnosis‐based models in R/R AML. Consistent with these findings, our results confirm the limited prognostic impact of *NPM1*, *IDH1/2* mutations in R/R setting.

This attenuation likely reflects clonal evolution and therapy pressure, suggesting new leukemogenic mechanisms driving progression independent of original mutations. In vitro data in *IDH1*‐mutant glioma show that *IDH1* acts as an early “driver” of tumorigenesis but shifts to a “passenger” role once transformation occurs. A similar mechanism may exist in AML biology, where *IDH1* and *NPM1* mutations, prognostically favorable in ND cases, lose significance at refractoriness or relapse [8].

These findings have important clinical implications. In AML, prognostication should rely on treatment context rather than solely on baseline mutations. Integrating genomic, MRD, clinical, and immune data may improve predictive accuracy. Evidence from a large machine learning study of 3062 AML cases indicates that the prognostic effect of mutations is highly context‐dependent. Favorable mutations such as *NPM1* or *CEBPA* show age‐related variability, whereas adverse ones like *TP53* or *ASXL1* remain unfavorable across age groups, supporting again a context‐ and treatment‐sensitive approach to risk stratification [9].

Although *NPM1* and *IDH1/2* mutations showed no prognostic benefit in our overall R/R cohort, emerging evidence indicates that some *IDH2*‐mutated AML patients can achieve durable responses to VEN‐HMA, especially when followed by allogeneic HSCT [10]. However, our study was not powered to evaluate post‐transplant outcomes, and only two of ten *IDH2*‐mutated patients underwent HSCT.

In conclusion, our data indicate that *NPM1* and *IDH1/2* mutations have limited prognostic relevance in R/R AML. Broader and standardized molecular profiling across centers will be essential to refine risk assessment, guide therapeutic decisions, and support enrollment in larger clinical or genomic studies.

## Conflicts of Interest

Giovanni Marconi: Honoraria, consulting or advisor from AbbVie Inc, Astellas Pharma, AstraZeneca, Enable life science, ImmunoGen, Janssen, Menarini Group, Pfizer, Ryvu Therapeutics, SERVIER, Syros Pharmaceuticals, Takeda; Speakers’ bureau: AbbVie Inc, Astellas Pharma, AstraZeneca, ImmunoGen, Janssen, Menarini Group, Pfizer, Ryvu Therapeutics, Syros Pharmaceuticals, Takeda; Research funding from AbbVie Inc, Pfizer, Jazz Pharmaceuticals, AstraZeneca, MEI Pharma, and Daiichi Sankyo. Calogero Vetro: Honoraria and advory board from AbbVie Inc, Astellas Pharma, Jazz Pharmaceuticals, BMS. Other authors have nothing to disclose.

## Supporting information


Supporting Information S1

## Data Availability

The data that support the findings of this study are available from the corresponding author upon reasonable request.
