# Supplementary material for: NPM1 and IDH1/2 Mutations Show Limited Prognostic Impact in Relapsed/Refractory AML: Evidence From the AVALON Cohort
Source: Hematol Oncol. 2026 Jan 13;44(1):e70169. doi: 10.1002/hon.70169 (PMC12797279; doi:10.1002/hon.70169)
Supplement: Supplementary file 1 — Supporting Information S1 [file HON-44-e70169-s001.docx]

# Supplementary Table S1. Comparison of clinical variables before VEN+HMA therapy in R/R AML patients tested for mutations in at least one gene among *IDH1*, *IDH2*, *NPM1*, *FLT3* (ITD or TKD)

|  | *Overall* | | | *IDH1* | | | *IDH2* | | | *NPM1* | | | *FLT3-*ITD | | | | *FLT3-*TKD | | |
| --- | --- | --- | --- | --- | --- | --- | --- | --- | --- | --- | --- | --- | --- | --- | --- | --- | --- | --- | --- |
|  | **not tested**  **(n=33)** | **tested**  **(n=114)** | **p** | **not tested**  **(n=97)** | **tested**  **(n=50)** | **p** | **not tested**  **(n=91)** | **tested**  **(n=56)** | **p** | **not tested**  **(n=56)** | **tested**  **(n=91)** | **p** | **not tested**  **(n=44)** | **tested**  **(n=103)** | **p** | **not tested**  **(n=92)** | | **tested**  **(n=55)** | **p** |
| Age – median [1Q‑3Q] (years) | 68 [60-72] | 62 [52-71] | 0.07 | 63 [53-71] | 66 [54-72] | 0.55 | 64 [55-71] | 64 [51-72] | 0.61 | 68 [57-72] | 63 [53-71] | 0.15 | 69 [63-74] | 61 [51-71] | 0.002 | 64 [55-72] | | 65 [52-71] | 0.58 |
| Age – n (%) |  |  |  |  |  |  |  |  |  |  |  |  |  |  |  |  | |  |  |
| ≤ 60 years | 9 (27) | 51 (45) |  | 41 (42) | 19 (38) |  | 35 (38) | 25 (45) |  | 19 (34) | 41 (45) |  | 9 (20) | 51 (50) |  | 34 (37) | | 26 (47) |  |
| > 60 years | 24 (73) | 63 (55) | 0.07 | 56 (58) | 31 (62) | 0.62 | 56 (62) | 31 (55) | 0.46 | 37 (66) | 50 (55) | 0.18 | 35 (80) | 52 (50) | 0.001 | 58 (63) | | 29 (53) | 0.22 |
| Sex – n (%) |  |  |  |  |  |  |  |  |  |  |  |  |  |  |  |  | |  |  |
| Female | 14 (42) | 48 (42) |  | 47 (48) | 15 (30) |  | 43 (47) | 19 (34) |  | 20 (36) | 42 (46) |  | 19 (43) | 43 (42) |  | 45 (49) | | 17 (31) |  |
| Male | 19 (58) | 66 (58) | 0.98 | 50 (52) | 35 (70) | 0.03 | 48 (53) | 37 (66) | 0.11 | 36 (64) | 49 (54) | 0.21 | 25 (57) | 60 (58) | 0.87 | 47 (51) | | 38 (69) | 0.03 |
| AML type – n (%) |  |  |  |  |  |  |  |  |  |  |  |  |  |  |  |  | |  |  |
| De novo AML | 21 (64) | 78 (68) |  | 70 (72) | 29 (58) |  | 66 (73) | 33 (59) |  | 32 (57) | 67 (74) |  | 28 (64) | 71 (69) |  | 56 (61) | | 43 (78) |  |
| Secondary AML | 10 (30) | 28 (25) |  | 21 (22) | 17 (34) |  | 20 (22) | 18 (32) |  | 17 (30) | 21 (23) |  | 14 (32) | 24 (23) |  | 29 (32) | | 9 (16) |  |
| Therapy-related AML | 2 (6) | 8 (7) | 0.85 | 6 (6) | 4 (8) | 0.21 | 5 (5) | 5 (9) | 0.23 | 7 (13) | 3 (3) | 0.045 | 2 (5) | 8 (8) | 0.50 | 7 (8) | | 3 (5) | 0.09 |
| Fitness – n (%) |  |  |  |  |  |  |  |  |  |  |  |  |  |  |  |  | |  |  |
| Fit | 20 (61) | 81 (71) |  | 69 (71) | 32 (64) |  | 65 (71) | 36 (64) |  | 39 (70) | 62 (68) |  | 27 (61) | 74 (72) |  | 62 (67) | | 39 (71) |  |
| Unfit to intensive CT | 13 (39) | 30 (26) |  | 25 (26) | 18 (36) |  | 24 (26) | 19 (34) |  | 16 (29) | 27 (30) |  | 16 (36) | 27 (26) |  | 29 (32) | | 14 (25) |  |
| Unfit to non-intensive CT | 0 | 3 (3) | 0.30 | 3 (3) | 0 | 0.24 | 2 (2) | 1 (2) | 0.71 | 1 (2) | 2 (2) | 1 | 1 (2) | 2 (2) | 0.43 | 1 (1) | | 2 (4) | 0.46 |
| ELN risk – n (%) |  |  |  |  |  |  |  |  |  |  |  |  |  |  |  |  | |  |  |
| Favorable | 0 | 7 (6) |  | 6 (8) | 1 (2) |  | 6 (8) | 1 (2) |  | 0 | 7 (8) |  | 3 (10) | 4 (4) |  | 5 (7) | | 2 (4) |  |
| Intermediate | 16 (76) | 56 (52) |  | 41 (51) | 31 (63) |  | 36 (49) | 36 (65) |  | 24 (56) | 48 (56) |  | 20 (65) | 52 (53) |  | 41 (54) | | 31 (58) |  |
| Adverse | 5 (24) | 45 (41) | 0.12 | 33 (41) | 17 (35) | 0.28 | 32 (43) | 18 (33) | 0.10 | 19 (44) | 31 (36) | 0.15 | 8 (26) | 42 (43) | 0.15 | 30 (39) | | 20 (38) | 0.80 |
| Pre-treatment hematologic values  – median [1Q-3Q] |  |  |  |  |  |  |  |  |  |  |  |  |  |  |  |  | |  |  |
| WBC (x10^9/L) | 2.9 [1.18-7.9] | 3.79 [1.6-16.1] | 0.63 | 3.3 [1.4-10.2] | 5.7 [1.3-16.8] | 0.68 | 3.4 [1.7-13] | 4.5 [1.0-11.4] | 0.79 | 3.4 [1.24-7.99] | 3.79 [1.8-16.8] | 0.69 | 3.5 [1.83-15] | 3.4 [1.37-11.87] | 0.53 | 3.8 [2.1-16.5] | | 2.6 [1.1-6.9] | 0.16 |
| Hgb (g/dL) | 8.85 [8.2-10] | 8.7 [8-9.8] | 0.61 | 8.95 [8.2-10] | 8.4 [7.85-9.7] | 0.13 | 8.9 [8.2-10] | 8.4 [7.9-9.8] | 0.26 | 8.9 [8.2-9.8] | 8.7 [8-9.95] | 0.60 | 8.7 [8.2-10.0] | 8.8 [8.2-9.8] | 0.82 | 8.9 [8.2-9.8] | | 8.6 [8-10.2] | 0.72 |
| PLT (x10^9/L) | 29.5 [16-73] | 49.5 [22-150] | 0.18 | 41.5 [16.5-143] | 37 [24-117] | 0.89 | 39 [16-127] | 38 [22-127] | 0.83 | 33 [23.5-116] | 44 [19-144.5] | 0.74 | 29.5 [14-63.5] | 57 [24-172.5] | 0.04 | 31 [16-94] | | 74 [26-173] | 0.08 |
| Bone marrow blasts (%) | 22 [9-50] | 30 [8-60] | 0.45 | 27 [9-53] | 35 [10-80] | 0.26 | 29 [9-55] | 30 [8-80] | 0.38 | 31 [10-60] | 29 [6-60] | 0.58 | 27 [10-63] | 30 [7-60] | 0.73 | 27 [10-60] | | 36 [7-60] | 0.97 |
| Best response to VEN+HMA – n (%) |  |  |  |  |  |  |  |  |  |  |  |  |  |  |  |  | |  |  |
| ORR – n (%) | 16 (55) | 52 (51) | 0.69 | 46 (53) | 22 (50) | 0.76 | 45 (55) | 23 (47) | 0.38 | 25 (53) | 43 (52) | 0.83 | 20 (56) | 48 (51) | 0.61 | 43 (54) | | 25 (49) | 0.60 |
| CR/CRi/CRp – n (%) | 13 (45) | 40 (39) |  | 36 (41) | 17 (39) |  | 35 (43) | 18 (37) |  | 21 (45) | 32 (38) |  | 17 (47) | 36 (38) |  | 31 (39) | | 22 (43) |  |
| PR – n (%) | 3 (10) | 12 (12) |  | 10 (11) | 5 (11) |  | 10 (12) | 5 (10) |  | 4 (9) | 11 (13) |  | 3 (8) | 12 (13) |  | 12 (15) | | 3 (6) |  |
| ^a^SD/PD – n (%) | 13 (45) | 50 (49) |  | 41 (47) | 22 (50) |  | 37 (45) | 26 (53) |  | 22 (47) | 41 (49) |  | 16 (44) | 47 (49) |  | 37 (46) | | 26 (51) |  |
| ED – n (%) | 4 (12) | 12 (10.5) |  | 10 (10) | 6 (12) |  | 9 (10) | 7 (13) |  | 9 (16) | 7 (8) |  | 8 (18) | 8 (8) |  | 12 (13) | | 4 (7) |  |
| Median time to best response (months) [1Q-3Q] | 2.2 [1.2-4.7] | 2.0 [1.1-3.7] | 0.67 | 2.1 [1.2-3.8] | 2.0 [1.0-4.3] | 0.95 | 2.1 [1.2-3.8] | 1.9 [1.0-4.3] | 0.74 | 2.3 [1.2-3.0] | 1.9 [1.0-4.3] | 0.95 | 2.2 [1.3-4.7] | 1.9 [1.0-3.5] | 0.29 | 1.7 [1.0-3.0] | | 2.9 [1.6-4.5] | 0.10 |
| Median DOR (months) [95% CI] | 5.1 [1.4-11.5] | 8.3 [6.3-11.9] | 0.19 | 9.3 [6.6-12.3] | 4.7 [3.0-6.5] | 0.02 | 9.3 [6.3-12.3] | 4.7 [2.6-8.3] | 0.01 | 5.1 [2.6-11.5] | 8.3 [6.3-13.2] | 0.09 | 6.9 [2.7-13.6] | 7.9 [4.5-11.2] | 0.71 | 8.3 [4.5-13.2] | | 6.5 [4.4-11.2] | 0.49 |
| Median number of prior lines [1Q-3Q] | 1 [1-2] | 2 [1-2] | 0.43 | 2 [1-2] | 1 [1-2] | 0.13 | 2 [1-3] | 1 [1-2] | 0.06 | 2 [1-2] | 2 [1-2] | 0.89 | 1 [1-3] | 2 [1-2] | 0.63 | 2 [1-2] | | 2 [1-2] | 0.55 |
| ^b^Median prior HMA cycles [1Q-3Q] | 8 [5-24] | 9 [4-14] | 0.42 | 7 [4-17] | 10 [6-16] | 0.44 | 7 [4-17] | 11 [6-16] | 0.33 | 9 [4-19] | 9 [4-14] | 0.48 | 8 [5-21] | 9 [4-14] | 0.24 | 7 [4-18] | | 10 [4-14] | 0.97 |
| Prior HMA – n (%) | 22 (67) | 33 (29) | <0.001 | 36 (37) | 19 (38) | 0.92 | 36 (40) | 19 (34) | 0.49 | 28 (50) | 27 (30) | 0.01 | 28 (64) | 27 (26) | <0.001 | 36 (39) | | 19 (35) | 0.58 |
| Prior intensive CT – n (%) | 17 (51) | 91 (80) | 0.001 | 73 (75) | 35 (70) | 0.49 | 68 (75) | 40 (71) | 0.66 | 36 (64) | 72 (79) | 0.048 | 24 (55) | 84 (82) | 0.001 | 66 (72) | | 42 (76) | 0.54 |
| HSCT before VEN+HMA – n (%) | 7 (21) | 13 (11) | 0.15 | 15 (15) | 5 (10) | 0.36 | 14 (15) | 6 (11) | 0.42 | 11 (20) | 9 (10) | 0.094 | 8 (18) | 12 (12) | 0.29 | 13 (14) | | 7 (13) | 0.81 |
| Type of HMA – n (%) |  |  |  |  |  |  |  |  |  |  |  |  |  |  |  |  | |  |  |
| 5-azacitidine + VEN | 26 (79) | 76 (67) |  | 70 (72) | 32 (64) |  | 66 (73) | 36 (64) |  | 37 (66) | 65 (71) |  | 35 (80) | 67 (65) |  | 69 (75) | | 33 (60) | 0.06 |
| Decitabine + VEN | 7 (21) | 38 (33) |  | 27 (28) | 18 (36) | 0.31 | 25 (27) | 20 (36) |  | 19 (34) | 26 (29) | 0.49 | 9 (20) | 36 (35) | 0.08 | 23 (25) | | 22 (40) | 0.06 |
| HMA pre-treatment – n (%) | 7 (21) | 38 (33) | 0.18 | 30 (31) | 15 (30) | 0.9 | 26 (29) | 19 (34) | 0.49 | 13 (23) | 32 (35) | 0.13 | 11 (25) | 34 (33) | 0.34 | 27 (29) | | 18 (33) | 0.67 |
| Prophylaxis – n (%) |  |  |  |  |  |  |  |  |  |  |  |  |  |  |  |  | |  |  |
| Antifungal prophylaxis | 6 (18) | 31 (27) | 0.29 | 22 (23) | 15 (30) | 0.33 | 21 (23) | 16 (29) | 0.46 | 14 (25) | 23 (25) | 0.97 | 7 (16) | 30 (29) | 0.09 | 25 (27) | | 12 (22) | 0.47 |
| Antibacterial prophylaxis | 4 (12) | 15 (13) | 1 | 12 (12) | 7 (14) | 0.80 | 11 (12) | 8 (14) | 0.80 | 12 (21) | 7 (8) | 0.02 | 5 (11) | 14 (14) | 0.71 | 10 (11) | | 9 (16) | 0.38 |
| Antiviral prophylaxis | 6 (18) | 22 (19) | 0.89 | 18 (19) | 10 (20) | 0.83 | 17 (18) | 11 (20) | 0.89 | 16 (29) | 12 (13) | 0.02 | 7 (16) | 21 (20) | 0.53 | 18 (20) | | 10 (18) | 0.84 |
| HSCT after VEN+HMA – n(%) | 6 (18) | 32 (28) | 0.36 | 22 (23) | 16 (32) | 0.31 | 21 (23) | 17 (30) | 0.43 | 11 (20) | 27 (30) | 0.29 | 7 (16) | 31 (30) | 0.13 | 20 (22) | | 18 (33) | 0.23 |

*Abbreviations: VEN = venetoclax; HMA = hypomethylating agents; CT = chemotherapy; HSCT = hematopoietic stem cell transplantation; CR = complete remission; CRi = complete remission with incomplete hematologic recovery; CRp = complete remission with incomplete platelet recovery; PR = partial response; SD = stable disease; PD = progressive disease; ORR = overall response rate (CR + CRi + CRp + PR); ED = early death (≤ 3 months without disease re-evaluation); DOR = duration of response; ELN = European LeukemiaNet; AML = acute myeloid leukemia; IDH1 = isocitrate dehydrogenase 1; IDH2 = isocitrate dehydrogenase 2; NPM1 = nucleophosmin 1; FLT3-ITD = FMS-like tyrosine kinase 3 – internal tandem duplication; FLT3-TKD = FMS-like tyrosine kinase 3 – tyrosine kinase domain; WBC = white blood cell count; Hgb = hemoglobin; PLT = platelet count; n = number (of patients); HSCT = Hematopoietic stem cell transplantation.*

*^a^ including patients who died within 3 months of starting VEN+HMA without disease re-evaluation; ^b^ patients who did not received prior HMA are excluded.*

# Supplementary Table S2. Clinical outcomes of R/R AML patients according to the mutational status of *IDH1*, *IDH2*, *NPM1*, *FLT3*-ITD and *FLT3*-TKD (mutated vs wild-type).

|  | *IDH1* | | | *IDH2* | | | *NPM1* | | | *FLT3-*ITD | | | *FLT3-*TKD | | |
| --- | --- | --- | --- | --- | --- | --- | --- | --- | --- | --- | --- | --- | --- | --- | --- |
|  | **wt**  **(n=47)** | **mut**  **(n=3)** | **p** | **wt**  **(n=46)** | **mut**  **(n=10)** | **p** | **wt**  **(n=79)** | **mut**  **(n=12)** | **p** | **wt**  **(n=89)** | **mut**  **(n=14)** | **p** | **wt**  **(n=53)** | **mut**  **(n=2)** | **p** |
| Best response – n (%) |  |  |  |  |  |  |  |  |  |  |  |  |  |  |  |
| CR/CRp/CRi | 17 (41) | 0 |  | 15 (37) | 3 (38) |  | 27 (37) | 5 (45) |  | 30 (36) | 6 (50) |  | 22 (44) | 0 |  |
| PR | 3 (7) | 2 (67) |  | 3 (7) | 2 (25) |  | 10 (14) | 1 (9) |  | 11 (13) | 1 (8) |  | 3 (6) | 0 |  |
| SD | 19 (46) | 1 (33) |  | 21 (51) | 3 (38) |  | 34 (47) | 4 (36) |  | 39 (47) | 4 (33) |  | 24 (48) | 1 (100) |  |
| ED | 2 (5) | 0 |  | 2 (5) | 0 |  | 2 (3) | 1 (9) |  | 3 (4) | 1 (8) |  | 1 (2) | 0 |  |
| ORR | 20 (44) | 2 (67) | 1 | 18 (41) | 5 (50) | 0.45 | 37 (48) | 6 (55) | 1 | 41 (48) | 7 (54) | 0.76 | 25 (49) | 0 | 1 |
| mDOR [95% CI], months | 4.7 [3.0–8.9] | 1.0 [1.0–NR] | 0.24 | 4.7 [2.0–8.9] | 6.5 [3.0–NR] | 0.87 | 8.3 [4.7–12.3] | 6.8 [4.4–NR] | 0.13 | 6.6 [4.4–12.3] | 10.7 [2.0–NR] | 0.66 | 6.5 [4.4–11.2] | -** | -** |
| mEFS [95% CI], months | 6.9 [4–9.7] | 2.4 [2.2–NR] | 0.37 | 5.4 [2.2–9.1] | 11.3 [1.4–14.3] | 0.51 | 6.3 [3.6–9.7] | 5.4 [0.8–NR] | 0.77 | 4.6 [3.2–7.4] | 8.4 [1.1–13.1] | 0.94 | 6.9 [3.2–9.8] | 6.2 [6.2–NR] | 0.88 |
| mOS [95% CI], months | 8.9 [4.4–12.2] | 2.4 [2.2–NR] | 0.18 | 6.2 [2.8–10.9] | 11.3 [1.4–14.3] | 0.56 | 9.1 [5.4–11.3] | 6 [1.6–31.2] | 0.72 | 6.3 [3.8–9.7] | 9.1 [3.2–13.2] | 0.74 | 9.5 [3.8–12.3] | 6.2 [6.2–NR] | 0.73 |
| HSCT after VEN+HMA – n(%) | 15 (32) | 1 (33) | 1 | 15 (33) | 2 (20) | 0.68 | 24 (30) | 3 (25) | 0.97 | 27 (30) | 4 (29) | 1 | 18 (34) | 0 | 0.81 |

*Abbreviations: wt = wild-type; mut = mutated; CR = complete remission; CRi = complete remission with incomplete hematologic recovery; CRp = complete remission with incomplete platelet recovery; PR = partial response; SD = stable disease; ED = early death (≤ 3 months without disease re-evaluation); ORR = overall response rate (CR + CRi + CRp + PR); DOR = duration of response; mDOR = median duration of response; mEFS = median event-free survival; mOS = median overall survival; NR = not reached; n = number of patients.*

*** not calculated because no response were observed.*

# Supplementary Table S3. Comparison of clinical variables before VEN+HMA therapy in ND AML patients tested for mutations in at least one gene among *IDH1*, *IDH2*, *NPM1*, *FLT3* (ITD or TKD).

|  | *Overall* | | | *IDH1* | | | *IDH2* | | | | *NPM1* | | | *FLT3-*ITD | | | *FLT3-*TKD | | |
| --- | --- | --- | --- | --- | --- | --- | --- | --- | --- | --- | --- | --- | --- | --- | --- | --- | --- | --- | --- |
|  | **not tested**  **(n=11)** | **tested**  **(n=32)** | **p** | **not tested**  **(n=32)** | **tested**  **(n=11)** | **p** | **not tested**  **(n=31)** | **tested**  **(n=12)** | **p** | **not tested**  **(n=18)** | | **tested**  **(n=25)** | **p** | **not tested**  **(n=18)** | **tested**  **(n=25)** | **p** | **not tested**  **(n=30)** | **tested**  **(n=13)** | **p** |
| Age – median [1Q‑3Q] (years) | 72 [67‑81] | 75 [68‑78] | 0.76 | 75 [68‑78] | 69 [63‑79] | 0.46 | 75 [67‑78] | 71 [0.66‑79] | 0.68 | 76 [68‑78] | | 73 [67‑77] | 0.12 | 77 [68‑78] | 73 [67‑77] | 0.07 | 74.5 [67‑77] | 73 [69‑78] | 0.73 |
| Age – n (%) |  |  |  |  |  |  |  |  |  |  | |  |  |  |  |  |  |  |  |
| ≤ 60 yr | 1 (9) | 3 (9) |  | 2 (6) | 2 (18) |  | 2 (6) | 2 (17) |  | 1 (6) | | 3 (12) |  | 1 (6) | 3 (12) |  | 2 (7) | 2 (15) |  |
| > 60 yr | 10 (91) | 29 (91) | 1 | 30 (94) | 9 (82) | 0.24 | 29 (94) | 10 (83) | 0.30 | 17 (94) | | 22 (88) | 0.47 | 17 (94) | 22 (88) | 0.47 | 28 (93) | 11 (85) | 0.37 |
| Sex – n (%) |  |  |  |  |  |  |  |  |  |  | |  |  |  |  |  |  |  |  |
| Female | 5 (45) | 18 (56) |  | 16 (50) | 7 (64) |  | 16 (52) | 7 (58) |  | 9 (50) | | 14 (56) |  | 8 (44) | 15 (60) |  | 17 (57) | 6 (46) |  |
| Male | 6 (55) | 14 (44) | 0.54 | 16 (50) | 4 (36) | 0.43 | 15 (48) | 5 (42) | 0.69 | 9 (50) | | 11 (44) | 0.70 | 10 (56) | 10 (40) | 0.31 | 13 (43) | 7 (54) | 0.53 |
| AML type – n (%) |  |  |  |  |  |  |  |  |  |  | |  |  |  |  |  |  |  |  |
| AML de novo | 2 (18) | 15 (47) |  | 13 (41) | 4 (36) |  | 12 (39) | 5 (42) |  | 5 (28) | | 12 (48) |  | 7 (39) | 10 (40) |  | 10 (33) | 7 (54) |  |
| AML secondary | 9 (82) | 15 (47) |  | 18 (56) | 6 (55) |  | 18 (58) | 6 (50) |  | 12 (67) | | 12 (48) |  | 11 (61) | 13 (52) |  | 20 (67) | 4 (31) |  |
| AML therapy related | 0 | 2 (6) | 0.13 | 1 (3) | 1 (9) | 0.71 | 1 (3) | 1 (8) | 0.75 | 1 (6) | | 1 (4) | 0.45 | 0 | 2 (8) | 0.67 | 0 | 2 (15) | 0.02 |
| Fitness – n (%) |  |  |  |  |  |  |  |  |  |  | |  |  |  |  |  |  |  |  |
| Fit | 3 (27) | 4 (13) |  | 6 (19) | 1 (9) |  | 6 (19) | 1 (8) |  | 4 (22) | | 3 (12) |  | 3 (17) | 4 |  | 5 (17) | 2 (15) |  |
| Unfit to intensive CT | 8 (73) | 27 (84) |  | 25 (78) | 10 (91) |  | 24 (77) | 11 (92) |  | 13 (72) | | 22 (88) |  | 15 (83) | 20 (80) |  | 25 (83) | 10 (77) |  |
| Unfit to non‑intensive CT | 0 | 1 (3) | 0.52 | 1 (3) | 0 | 0.74 | 1 (3) | 0 | 0.75 | 1 (6) | | 0 | 0.29 | 0 | 1 (4) | 1 | 0 | 1 (8) | 0.43 |
| ELN risk – n (%) |  |  |  |  |  |  |  |  |  |  | |  |  |  |  |  |  |  |  |
| Favorable | 0 | 6 (20) |  | 4 (17) | 2 (18) |  | 4 (17) | 2 (17) |  | 0 | | 6 (26) |  | 3 (25) | 3 (13) |  | 5 (23) | 1 (8) |  |
| Intermediate | 3 (60) | 16 (53) |  | 12 (50) | 7 (64) |  | 11 (48) | 8 (67) |  | 7 (58) | | 12 (52) |  | 7 (58) | 12 (52) |  | 8 (36) | 11 (85) |  |
| Adverse | 2 (40) | 8 (27) | 0.68 | 8 (33) | 2 (18) | 0.63 | 8 (35) | 2 (17) | 0.57 | 5 (42) | | 5 (22) | 0.12 | 2 (17) | 8 (35) | 0.45 | 9 (41) | 1 (8) | 0.02 |
| Pre-treatment hematologic values –  median [1Q-3Q] |  |  |  |  |  |  |  |  |  |  | |  |  |  |  |  |  |  |  |
| WBC (×10⁹/L) | 6.6 [3.3‑21] | 6 [2.7‑44.3] | 0.85 | 6.6 [3.0‑23.8] | 4.6 [1.4‑43.60] | 0.75 | 6.5 [2.8‑26.7] | 4.8 [1.4‑43.60] | 0.95 | 8.5 [4‑19.3] | | 4.7 [1.9‑44.3] | 0.52 | 7.6 [4.0‑23.8] | 4.8 [1.9‑44.3] | 0.44 | 6 [3.3‑38.2] | 7.5 [1.9‑19.3] | 0.82 |
| Hgb (g/dL) | 9.8 [8.1‑12] | 9.2 [8.5‑10.8] | 0.54 | 9.6 [8.6‑12] | 8.9 [7.8‑9.3] | 0.05 | 9.4 [8.6‑12] | 9 [7.8‑9.7] | 0.19 | 9.3 [8.6‑11.6] | | 9.2 [8.5‑10.8] | 0.60 | 9.8 [8.8‑12] | 9 [8.4‑10.5] | 0.10 | 9 [8.3‑11.4] | 9.7 [9.2‑12] | 0.18 |
| PLT (×10⁹/L) | 31 [11‑62] | 44 [13‑97] | 0.38 | 47 [18.5‑85] | 29.5 [12‑84] | 0.65 | 50 [18‑92] | 31 [12‑84] | 0.68 | 44 [19‑75] | | 41 [13‑97] | 0.75 | 37.5 [12‑69.5] | 45.5 [18‑92] | 0.45 | 31 [11‑77] | 71 [39‑98] | 0.11 |
| Bone‑marrow blasts % | 25.5 [20‑37] | 46 [20‑75] | 0.06 | 45 [21‑70] | 31 [17‑47] | 0.23 | 42.5 [20.5‑69.5] | 31.5 [18.5‑61] | 0.53 | 32 [21‑58] | | 45 [20‑70] | 0.73 | 29 [20‑51.5] | 46 [25.5‑75] | 0.14 | 32 [20‑58] | 50 [32‑80] | 0.06 |
| Best response – n (%) |  |  |  |  |  |  |  |  |  |  | |  |  |  |  |  |  |  |  |
| ORR | 7 (64) | 21 (72) | 0.59 | 22 (71) | 6 (67) | 0.80 | 22 (73) | 6 (60) | 0.43 | 11 (65) | | 17 (74) | 0.53 | 12 (71) | 16 (70) | 0.94 | 20 (71) | 8 (67) | 0.76 |
| CR/CRi/CRp | 3 (27) | 18 (62) |  | 18 (58) | 3 (33) |  | 18 (60) | 3 (30) |  | 7 (41) | | 14 (61) |  | 8 (47) | 13 (57) |  | 15 (54) | 6 (50) |  |
| PR | 4 (36) | 3 (10) |  | 4 (13) | 3 (33) |  | 4 (13) | 3 (30) |  | 4 (24) | | 3 (13) |  | 4 (24) | 3 (13) |  | 5 (18) | 2 (17) |  |
| ^a^SD/PD | 4 (36) | 8 (28) |  | 9 (29) | 3 (33) |  | 8 (27) | 4 (40) |  | 6 (35) | | 6 (26) |  | 5 (29) | 7 (30) |  | 8 (29) | 4 (33) |  |
| ED | 0 | 3 (9) |  | 1 (3) | 2 (18) |  | 1 (3) | 2 (16) |  | 1 (5) | | 2 (8) |  | 1 (6) | 2(8) |  | 2 (7) | 1 (8) |  |
| Median TTR (mo) [1Q‑3Q] | 2.6 [1.7‑3.7] | 4.0 [1.3‑8.3] | 0.63 | 3.4 [1.7‑6.7] | 1.5 [1.2‑4.0] | 0.24 | 3.4 [1.7‑6.7] | 1.5 [1.2‑4.0] | 0.24 | 2.6 [1.7‑3.9] | | 4.0 [1.3‑8.3] | 0.76 | 2.8 [1.6‑8.6] | 3.2 [1.3‑5.9] | 0.80 | 3.4 [1.6‑6.7] | 1.7[1.0‑5.3] | 0.26 |
| Median DOR (mo) [95% CI] | 10.6 [3.0‑NR] | 11.9 [3.2‑NR] | 0.16 | 10.9 [4.0‑11.9] | 7.6 [1.9‑NR] | 0.78 | 10.9 [4.0‑11.9] | 7.6 [1.9‑NR] | 0.78 | 10.6 [3.0‑NR] | | 11.9 [1.9‑NR] | 0.46 | 10.6 [3.0‑11.9] | 11.9 [1.9‑NR] | 0.46 | 10.9 [3.2‑13.1] | 7.6 [0.8‑NR] | 0.67 |
| Median EFS (mo) [95% CI] | 5 [0.4‑12.5] | 9.2 [4.5‑20.1] | 0.07 | 7.2 [2.6‑20.1] | 5.9 [2.2‑12.7] | 0.35 | 12.3 [3‑20.1] | 5.7 [1.7‑12.7] | 0.20 | 5.1 [1.6‑12.5] | | 12.3 [3.1‑20.1] | 0.33 | 5.3 [1.6‑15.6] | 9.2 [3‑18.6] | 0.90 | 7.7 [3‑20.1] | 5.7 [1.9‑12.7] | 0.29 |
| Median OS (mo) [95% CI] | 7.1 [0.4‑12.5] | 15.8 [6.5‑25.6] | 0.02 | 15.1 [5.1‑32] | 9.2 [4.5‑17.7] | 0.25 | 15.6 [5.1‑32] | 9.2 [4.5‑17.7] | 0.24 | 7.7 [2.7‑15.6] | | 14 [5.7‑25.6] | 0.28 | 7.7 [5.1‑15.8] | 12.7 [4.5‑19.4] | 0.85 | 7.7 [4.5‑25.6] | 14 [5.7‑17.7] | 0.45 |

*Abbreviations: VEN = venetoclax; HMA = hypomethylating agents; CT = chemotherapy; HSCT = hematopoietic stem cell transplantation; CR = complete remission; CRi = complete remission with incomplete hematologic recovery; CRp = complete remission with incomplete platelet recovery; PR = partial response; SD = stable disease; PD = progressive disease; ORR = overall response rate (CR + CRi + CRp + PR); ED = early death (≤ 3 months without disease re-evaluation); DOR = duration of response; TTR = time-to-response; EFS = event-free survival; OS = overall survival; ELN = European LeukemiaNet; AML = acute myeloid leukemia; IDH1 = isocitrate dehydrogenase 1; IDH2 = isocitrate dehydrogenase 2; NPM1 = nucleophosmin 1; FLT3-ITD = FMS-like tyrosine kinase 3 – internal tandem duplication; FLT3-TKD = FMS-like tyrosine kinase 3 – tyrosine kinase domain; WBC = white blood cell count; Hgb = hemoglobin; PLT = platelet count; n = number (of patients); NR = not reached.*

*^a^ including patients who died within 3 months of starting VEN+HMA without disease re-evaluation.*

**Supplementary Table S4.** Clinical outcomes of ND AML patients according to the mutational status of *IDH1*, *IDH2*, *NPM1*, *FLT3-*ITD and *FLT3-*TKD (mutated vs wild-type).

|  | *IDH1* | | | *IDH2* | | | *NPM1* | | | *FLT3-*ITD | | | *FLT3-*TKD | | |
| --- | --- | --- | --- | --- | --- | --- | --- | --- | --- | --- | --- | --- | --- | --- | --- |
|  | **wt**  **(n=10)** | **mut**  **(n=1)** | **p** | **wt**  **(n=9)** | **mut**  **(n=3)** | **p** | **wt**  **(n=18)** | **mut**  **(n=7)** | **p** | **wt**  **(n=22)** | **mut**  **(n=3)** | **p** | **wt**  **(n=11)** | **mut**  **(n=2)** | **p** |
| Best response – n (%) |  |  |  |  |  |  |  |  |  |  |  |  |  |  |  |
| CR/CRp/CRi | 2 (25) | 1 (100) |  | 1 (14) | 2 (67) |  | 8 (47) | 6 (86) |  | 10 (50) | 3 (100) |  | 5 (50) | 1 (50) |  |
| PR | 3 (38) | 0 |  | 3 (43) | 0 |  | 3 (18) | 0 |  | 3 (15) | 0 |  | 2 (20) | 0 |  |
| SD | 3 (38) | 0 |  | 3 (43) | 1 (33) |  | 6 (35) | 0 |  | 7 (35) | 0 |  | 3 (30) | 1 (50) |  |
| ED | 2 (20) | 0 |  | 2 (22) | 0 |  | 1 (6) | 1 (14) |  | 2 (9) | 0 |  | 1 (9) | 0 |  |
| ORR – n (%) | 5 (63) | 1 (100) | 1 | 4 (44) | 2 (67) | 1 | 11 (61) | 6 (100) | 0.14 | 13 (59) | 3 (100) | 0.53 | 7 (64) | 1 (50) | 1 |
| mDOR [95% CI] | 7.6 [1.9–NR] | NA | NA | 3.2 [1.9-NR] | NR] | 0.30 | 4.0 [0.8-NR] | 13.1 [13.1-NR] | 0.02 | 7.6 [0.8-NR] | NR | 0.08 | 11.9 [0.8-NR] | NR [NR-NR] | 0.40 |
| mEFS [95% CI] | 5.7 [1.7-9.2] | NR | 0.09 | 5.7 [1.7-9.2] | 12.7 [1.6-NR] | 0.23 | 5.7 [2.5-12.7] | NR [3.1-NR] | 0.01 | 5.7 [2.5-12.7] | 43.1 [NR-NR] | 0.02 | 5.9 [1.9-18.6] | 1.6 [1.6-NR] | 0.11 |
| mOS [95% CI] | 7.7 [1.7-14] | NR | 0.09 | 7.7 [1.7-14] | 17.7 [15.1-NR} | 0.06 | 9.2 [4.5-17.7] | NR [3.1-NR] | 0.03 | 9.2 [3.1-18.6] | 43.1 [NR-NR] | 0.02 | 12.7 [2.7-18.6] | 15.1 [15.1-NR] | 1 |

*Abbreviations: wt = wild-type; mut = mutated; CR = complete remission; CRi = complete remission with incomplete hematologic recovery; CRp = complete remission with incomplete platelet recovery; PR = partial response; SD = stable disease; ED = early death (≤ 3 months); ORR = overall response rate (CR + CRi + CRp + PR); ED = early death (≤ 3 months without disease re-evaluation); DOR = duration of response; mDOR = median duration of response; mEFS = median event-free survival; mOS = median overall survival; NR = not reached; n = number of patients; NA = not available*

# Supplementary Table S5. Clinical outcomes in ND AML by *NPM1*/*IDH1*/*IDH2* mutational status: response, duration of response, event-free survival, and overall survival in mutated vs. triple-wild-type patients.

|  | *NPM1/IDHs* | |  |
| --- | --- | --- | --- |
|  | **wt**  **(n=7)** | **mut**  **(n=9)** | ***p*** |
| Best response – n (%) |  |  |  |
| CR/CRp/CRi | 0 | 7 (88) |  |
| PR | 3 (50) | 0 |  |
| SD | 3 (50) | 1 (13) |  |
| ED | 1 (14) | 1 (11) |  |
| ORR – n (%) | 3 (50) | 7 (88) | 0.12 |
| mDOR [95% CI] | 3.2 [1.9-NR] | 13.1 [13.1-NR] | 0.01 |
| mEFS [95% CI] | 5.1 [1.7-5.9] | 20.1 [1.6-NR] | 0.006 |
| mOS [95% CI] | 6.3 [1.7-12.7] | 25.6 [3.1-NR] | <0.001 |

*Abbreviations: wt = wild-type; mut = mutated; CR = complete remission; CRi = complete remission with incomplete hematologic recovery; CRp = complete remission with incomplete platelet recovery; PR = partial response; SD = stable disease; ED = early death (≤ 3 months); ORR = overall response rate (CR + CRi + CRp + PR); DOR = duration of response; mDOR = median duration of response; mEFS = median event-free survival; mOS = median overall survival; NR = not reached; n = number of patients*

**Figure S1:** Event free survival (A) and overall survival (B) in newly diagnosed AML receiving VEN+HMAs comparing those with wild-type *NPM1* and *IDH1/2* (triple-wild-type) versus those with at least one mutation in *NPM1*, *IDH1*, or *IDH2*.


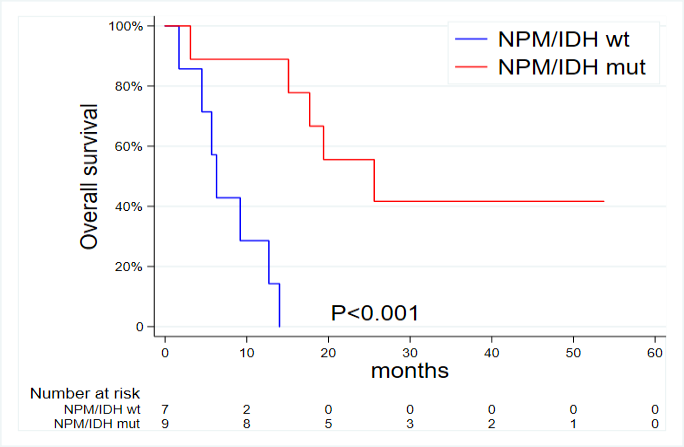

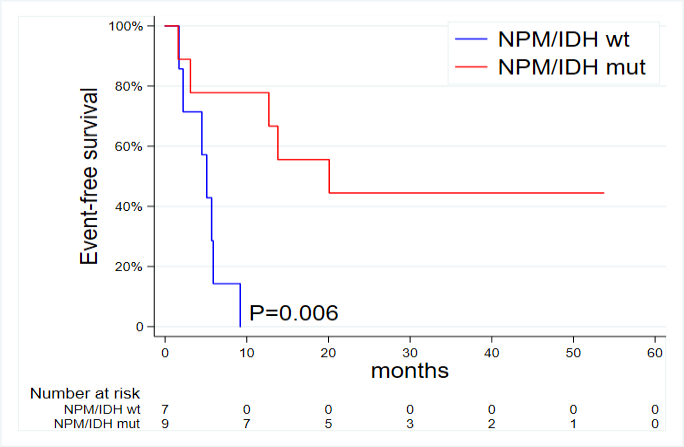


**A**

**B**

**Figure S2**: Kaplan–Meier curves comparing event-free survival (A) and overall survival (B) in patients carrying at least one *NPM1* or *IDH1/2* mutation, stratified by treatment setting. Patients treated in the newly diagnosed (ND) setting (red line) exhibited significantly improved survival compared to those treated in the relapsed/refractory (R/R) setting (blue line).

**
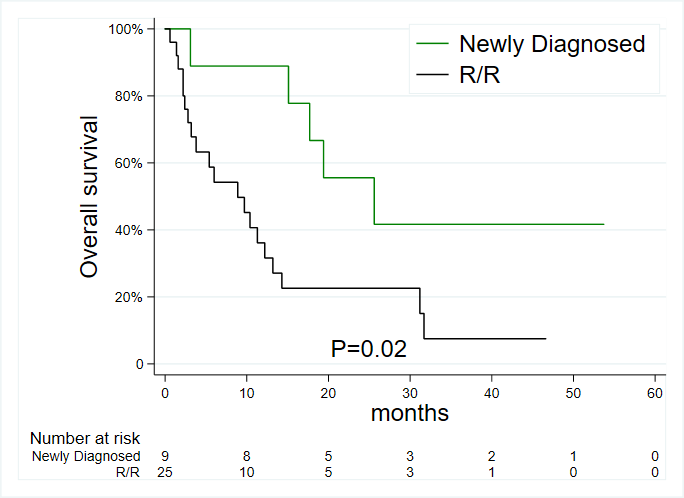

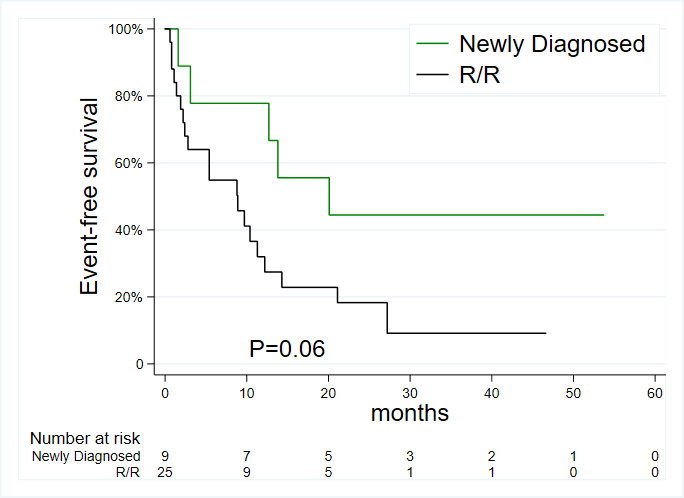
**

**A**

**B**

**Supplementary Table S6.** AVALON Cooperative Group

| **Contributor** | **Affiliation** |
| --- | --- |
| Adriano Venditti | U.O.C. Ematologia, A.O.U. Fond. Policlinico Tor Vergata |
| Agostino Tafuri | U.O.C. Ematologia, A.O.U. Sant'Andrea, Roma |
| Alessandra Romano | A.O.U. Policlinico Vittorio Emanuele, Catania |
| Alessandro Cignetti | Divisione Universitaria di Ematologia e Terapie Cellulari, A.O. Ordine Mauriziano, Torino |
| Alfredo Molteni | U.O.C. Ematologia – CTMO, ASST Cremona, Cremona |
| Annalisa Imovilli | Dip. Oncologico e Tecnologie avanzate, IRCCS Arcispedale S. Maria Nuova, Reggio Emilia |
| Bianca Serio | A.O.U. S. Giovanni di Dio e Ruggi d'Aragona, Università di Salerno, Salerno |
| Bruna Messere | Dip. Oncopneumoematologico, A.O.R.N. “A. Cardarelli”, Napoli |
| Calogero Vetro | Hematology and Bone Marrow Transplantation Unit, Hospital of Bolzano (SABES-Azienda Sanitaria dell’Alto Adige), Teaching Hospital of Paracelsus Medical University, Bolzano, Italy |
| Carla Mazzone | Dep. Hematology S. Eugenio Ospital, Roma |
| Carmine Selleri | A.O.U. S. Giovanni di Dio e Ruggi d'Aragona, Università di Salerno, Salerno |
| Chiara Zingaretti | IRCCS Istituto Romagnolo per lo studio dei Tumori “Dino Amadori” - IRST S.r.l., Meldola |
| Claudia Basilico | ASST-Settelaghi, Ospedale di Circolo-Fondazione Macchi, Varese |
| Claudio Cerchione | IRCCS Istituto Romagnolo per lo studio dei Tumori “Dino Amadori” - IRST S.r.l., Meldola |
| Corrado Tarella | Div. Oncoematologia, European Institute of Oncology, Milano |
| Cristina Papayannidis | Dip. di Medicina Specialistica, Diagnostica e Sperimentale, Università di Bologna, Bologna |
| Daniela Cilloni | Department of Clinical and Biological Sciences, University of Turin |
| Daniele Mattei | S. C. Ematologia, A.S.O. S. Croce e Carle, Cuneo |
| Davide Griguolo | S. C. Ematologia, A.O.U. Giuliano Isontina, Trieste |
| Elisa Roncoroni | Department of Hematology Oncology, Fondazione IRCCS Policlinico San Matteo, Pavia |
| Elisabetta Abruzzese | Dep. Hematology S. Eugenio Ospital, Roma |
| Elisabetta Petracci | IRCCS Istituto Romagnolo per lo studio dei Tumori “Dino Amadori” - IRST S.r.l., Meldola |
| Elisabetta Todisco | Div. Oncoematologia, European Institute of Oncology, Milano |
| Endri Mauro | Azienda U.L.S.S.9 Ospedale Regionale Cà Foncello, Treviso |
| Erika Borlenghi | Department of Hematology, ASST Spedali Civili di Brescia, Brescia |
| Ernesta Audisio | SC Ematologia 2, Dip. di Ematologia e Oncologia, A.O.U. Città della Salute e della Scienza, Torino |
| Fabio Ciceri | U.O. Ematologia e TMO, Ospedale S.Raffaele, Milano |
| Federica Gigli | Div. Oncoematologia, European Institute of Oncology, Milano |
| Federica Monaco | AUSL della Romagna, Ospedale S. Maria delle Croci, Ravenna |
| Federico Lussana | Department of Oncology and Hematology University of Milan, and Azienda SocioSanitaria Territoriale Papa Giovanni XXIII, Bergamo |
| Felicetto Ferrara | Dip. Oncopneumoematologico, A.O.R.N. “A. Cardarelli”, Napoli |
| Flavia Rivellini | Osp. “A. Tortora” di Pagani, Pagani |
| Francesco Di Raimondo | A.O.U. Policlinico Vittorio Emanuele, Catania |
| Francesco Lanza | AUSL della Romagna, Ospedale S. Maria delle Croci, Ravenna |
| Francesco Zaja | S. C. Ematologia, A.O.U. Giuliano Isontina, Trieste |
| Giorgio Priolo | SC Ematologia 2, Dip.di Ematologia e Oncologia, AOU Città della Salute e della Scienza, Torino |
| Giovanni Marconi | IRCCS Istituto Romagnolo per lo studio dei Tumori “Dino Amadori” - IRST S.r.l., Meldola |
| Giovanni Martinelli | IRCCS Istituto Romagnolo per lo studio dei Tumori “Dino Amadori”, IRST S.r.l., Meldola |
| Giuliana Rizzuto | Department of Oncology and Hematology University of Milan, and Azienda SocioSanitaria Territoriale Papa Giovanni XXIII, Bergamo |
| Giuseppe Rossi | Department of Hematology, ASST Spedali Civili di Brescia, Brescia |
| Serena Luponio | A.O.U. S. Giovanni di Dio e Ruggi d'Aragona, Università di Salerno, Salerno |
| Ilenia Manfra | A.O.R.N. “S. Giuseppe Moscati”, Avellino |
| Irene Urbino | SC Ematologia 2, Dipartmento di Ematologia e Oncologia, AOU Città della Salute e della Scienza, Torino |
| Irene Valli | IRCCS Istituto Romagnolo per lo studio dei Tumori “Dino Amadori” - IRST S.r.l., Meldola |
| Jacopo Nanni | Dip. di Medicina Specialistica, Diagnostica e Sperimentale, Università di Bologna, Bologna |
| Katia Codeluppi | Dip. Oncologico e Tecnologie avanzate, IRCCS Arcispedale S. Maria Nuova, Reggio Emilia |
| Liliana Calabrese | Div. Oncoematologia, European Institute of Oncology, Milano |
| Luana Fianchi | Istituto di Ematologia, Fondazione Policlinico Universitario “A. Gemelli” IRCCS, Roma |
| Luca Facchini | Dip. Oncologico e Tecnologie avanzate, IRCCS Arcispedale S. Maria Nuova, Reggio Emilia |
| Luca Maurillo | U.O.C. Ematologia, A.O.U. Fond. Policlinico Tor Vergata |
| Maria Paola Martelli | Department of Medicine and Surgery, Perugia University, "Santa Maria della Misericordia" Hospital, Perugia |
| Maria Benedetta Giannini | IRCCS Istituto Romagnolo per lo studio dei Tumori “Dino Amadori” - IRST S.r.l., Meldola |
| Maria Chiara Abbenante | Dip. Onco-ematologia IRCCS Casa Sollievo della Sofferenza, S.Giovanni Rotondo (FG) |
| Mariarita Sciumè | U.O.C. Oncoematologia, Fond. IRCCS Ca' Granda Ospedale Maggiore Policlinico di Milano, Milano |
| Michele Gottardi | Azienda U.L.S.S.9 Ospedale Regionale Cà Foncello, Treviso |
| Michelina Dargenio | Hematology and SCT Unit, “Vito Fazzi” Hospital, Lecce |
| Monia Lunghi | Divisione di Ematologia, Dip.di Medicina Traslazionale, Università del Piemonte Orientale, Novara |
| Monica Fumagalli | Divisione di Ematologia Osp. S. Gerardo ASST Monza, Monza |
| Nicola Cascavilla | Dip. Onco-ematologia IRCCS Casa Sollievo della Sofferenza, S.Giovanni Rotondo (FG) |
| Nicola Di Renzo | Hematology and SCT Unit, “Vito Fazzi” Hospital, Lecce |
| Nicola Stefano Fracchiolla | U.O.C. Oncoematologia, Fond. IRCCS Ca' Granda Ospedale Maggiore Policlinico di Milano, Milano |
| Paolo De Fabritis | Dep. Hematology S. Eugenio Ospital, Roma |
| Pasquale De Roberto | U.O.C. Oncoematologia, Fond. IRCCS Ca' Granda Ospedale Maggiore Policlinico di Milano, Milano |
| Patrizia Zappasodi | Department of Hematology Oncology, Fondazione IRCCS Policlinico San Matteo, Pavia |
| Prassede Salutari | Dipartimento di Ematologia e Madicina Trasfusionale – Ospedale Civile di Pescara, Pescara |
| Raffaele Palmieri | U.O.C. Ematologia, A.O.U. Fond. Policlinico Tor Vergata |
| AnnaMaria Della Corte | A.O.U. S. Giovanni di Dio e Ruggi d'Aragona, Università di Salerno, Salerno |
| Roberta Volpi | IRCCS Istituto Romagnolo per lo studio dei Tumori “Dino Amadori” - IRST S.r.l., Meldola |
| Roberto Cairoli | SC Ematologia, ASST Grande Ospedale Metropolitano Niguarda, Milano |
| Simona Menna | Div. Oncoematologia, European Institute of Oncology, Milano |
| Sofia Sciabolacci | Department of Medicine – Section of Hematology and Clinical Immunology, Perugia University, "Santa Maria della Misericordia" Hospital, Perugia |
| Susanna Gallo | Divisione Universitaria di Ematologia e Terapie Cellulari, A.O. Ordine Mauriziano, Torino |
| Valentina Oliva | Dip. Oncopneumoematologico, A.O.R.N. “A. Cardarelli”, Napoli |
| Valeria Cardinali | Department of Medicine and Surgery, Perugia University, "Santa Maria della Misericordia" Hospital, Perugia |
| Vincenza Martini | U.O.C. Ematologia, Osp. F. Spaziani, Frosinone |
| Vincenzo Federico | Hematology and SCT Unit, “Vito Fazzi” Hospital, Lecce |
| Viviana Amato | Div. Oncoematologia, European Institute of Oncology, Milano |
